# Experimental Analysis of the Effects of Image Lightness and Chroma Modulation on the Reproduction of Glossiness, Transparency and Roughness

**DOI:** 10.3390/jimaging12040159

**Published:** 2026-04-08

**Authors:** Hideyuki Ajiki, Midori Tanaka

**Affiliations:** 1 Graduate School of Science and Engineering, Chiba University, Yayoi-cho 1-33, Inage-ku, Chiba 263-8522, Japan; 2 Graduate School of Informatics, Chiba University, Yayoi-cho 1-33, Inage-ku, Chiba 263-8522, Japan

**Keywords:** appearance, glossiness, transparency, roughness, image reproduction

## Abstract

Even when an object’s color is accurately reproduced in a colorimetrically reproduced image (CRI), the perceived material appearance does not necessarily match that of the original object. This mismatch remains a challenge for faithfully reproducing real-world appearance in digital media. In this study, we investigated how lightness and chroma modulation affect the perception of glossiness, transparency, and roughness. These three attributes were quantitatively correlated with physical surface properties and image features through a direct comparison between objects and images. Observers selected the images that best matched the material appearance of the physical samples for each attribute. Image features derived from the gray-level co-occurrence matrix (GLCM) and surface roughness parameters were analyzed to compare the selected images with the CRI. In the lightness experiment, observers consistently selected images with higher lightness than the CRI, which was accompanied by increased complexity in the luminance distribution. In the chroma experiment, images with higher chroma were preferred; however, changes in GLCM features were negligible. Notably, stimuli with small local luminance differences at the CRI required larger shifts in image features to achieve perceptual matching. These findings indicate that modulating the luminance distribution is crucial for aligning the perceived appearance between physical objects and their digital representations.

## 1. Introduction

Humans perceive color and material appearance based on visual information produced by the interplay between the physical surface properties of objects and the illumination environment. Material appearance perception is a fundamental factor influencing affective evaluation and value judgment, and systematic research has accumulated in fields such as information engineering, psychophysics, and neuroscience. In particular, glossiness, transparency, and roughness—resulting from surface reflection, transmission, and micro-scale surface irregularities—have been extensively studied as key indices in appearance design and product development.

Ferwerda et al. [1] quantitatively modeled the relationship between physical reflectance properties and perceptual dimensions of gloss based on Hunter’s gloss classification [2]. They demonstrated that gloss perception is primarily structured along the dimensions of contrast gloss (the luminance ratio of specular to diffuse reflection) and distinctness-of-image (DOI), defined as the sharpness of the reflected image, and reported that DOI exhibits a linear relationship with surface roughness. Beuckels et al. [3] further examined the influence of haze, arising from surface scattering, within an existing gloss space and showed that, for physical samples including haze, observers may rely on highlight brightness and contrast as cues for gloss perception. Chen et al. [4] demonstrated a perceptual discrepancy in glossiness between physical objects and display images. They reported that this discrepancy can increase under diffuse illumination or reduced display luminance, but can decrease under spotlight illumination, where highlights serve as effective perceptual cues. Regarding the cues used by the visual system for material estimation, Fleming et al. [5] showed that observers utilize prior knowledge of illumination statistics, leading to stable reflectance estimation under natural illumination but reduced accuracy under artificial illumination lacking statistical regularity. Their findings suggest an estimation process based on image statistics rather than strict inverse optics. Motoyoshi et al. [6] established that simple image statistics, such as luminance histogram skewness, can serve as cues for judgments of glossiness and lightness. Tanaka et al. [7] reported that gloss perception cannot always be sufficiently explained by specular gloss units (GUs) alone. They showed that a predictive model incorporating GU, haze, DOI, and gray-level co-occurrence matrix (GLCM) features of luminance images achieved higher accuracy than GU alone. Furthermore, Tian et al. [8] showed, using facial images, that increased physical roughness reduces perceived glossiness and that increased glossiness may be associated with decreased perceived lightness. Hansmann-Roth et al. [9] investigated gloss perception through a transparent layer and established that contrast reduction caused by the transparent layer influences gloss judgments.

Fleming et al. [10] differentiated between transparency and translucency, illustrating that perceived translucency can vary substantially depending on the direction of illumination. Motoyoshi et al. [11] treated transparency as a continuous dimension ranging from opaque to highly transparent and suggested that the preservation of highlights and contrast inconsistency in non-specular components may serve as cues for transparency perception. Furthermore, Gigilashvili et al. [12] examined transparency and opacity as a continuous dimension and tested the hypothesis that translucency reaches a maximum at an intermediate region, reporting that this tendency varies depending on object shape.

Regarding roughness perception, Ho et al. [13] showed that roughness constancy can break down under changes in illumination direction and reported that illumination-dependent cues, such as shadow proportion and luminance variability, strongly influence roughness judgments. Delanoy et al. [14], through comparisons between paintings and rendered images, demonstrated that material appearance can be explained by a limited number of image cues, such as highlight sharpness and contrast, even across different media. With respect to haptic roughness perception, Lee et al. [15] analyzed the relationship between subjective evaluations and physical surface properties, including friction coefficient and arithmetic mean roughness, for synthetic fibers, and reported the feasibility of prediction using machine learning. Tanaka et al. [16] experimentally analyzed perceived glossiness, transparency, and roughness for physical objects and for images with manipulated color and resolution, as well as changes in perceived material appearance between object and image conditions. Their results showed that perceived glossiness and transparency in images can be weaker than in physical objects. They also reported that, even among images of the same object, lower-resolution images were perceived as rougher for certain samples. These findings suggest that presentation conditions, such as digital imaging and image resolution, as well as material properties, can influence perceived material appearance.

Prior research indicates that material perception depends not directly on physical quantities, but strongly on image features such as contrast, highlights, luminance statistics, and spatial frequency. However, a systematic framework that directly compares physical objects with diverse surface properties and their corresponding images and explains perceptual differences in material appearance based on both physical surface parameters and image features has not been adequately developed. In our previous work, perceived glossiness, transparency, and roughness were analyzed through direct comparisons between physical objects and images whose colors were colorimetrically reproduced based on tristimulus values [17]. The results showed that perceived glossiness and transparency in images tend to be less than those of physical objects, whereas perceived roughness in images can be either stronger or weaker depending on the sample. Furthermore, using the method proposed by Manabe et al. [18,19], the specular reflection component and spatial frequency components of the CRIs were systematically modulated and experiments were conducted in which observers selected images perceived to match the glossiness or roughness of the physical objects. The findings suggested that adjusting image features derived from the GLCM, such as contrast and angular second moment, may be effective for reproducing glossiness and roughness in images. Nonetheless, the simultaneous variation in several image attributes under those conditions complicated the identification of the primary factors influencing material reproduction. Therefore, in the current study, image sets were generated by stepwise modulation of CIE 1976 L* and C* values based on the CRI. Through direct comparison experiments with physical objects, observers selected images perceived to match glossiness, transparency, and roughness. By analyzing the image features of the selected images, the features contributing to perceptual matching were identified and the required magnitude of change was quantified. Furthermore, by examining relationships between physical surface roughness parameters and image features, the factors necessary to align perceived material appearance between physical objects and images were clarified.

## 2. Materials and Methods

### 2.1. Real Stimulus

**Figure 1:**
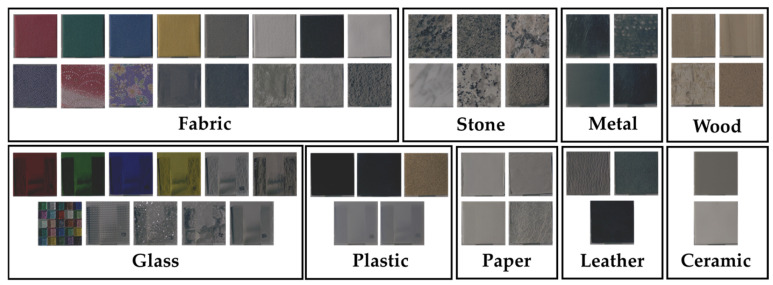
Experimental stimuli.

In this study, to analyze perceived material appearance between physical objects and images without relying on material-specific characteristics, physical stimuli were selected from everyday materials with diverse colors and surface properties. A total of 55 physical samples were prepared, representing nine material categories: fabric, plastic, glass, stone, ceramic, metal, leather, wood, and paper. To minimize the influence of shape cues on material perception, all samples were standardized as square plates measuring 50 mm on each side. Figure 1 shows the physical stimuli used in this study.

### 2.2. Image Stimulus

Image stimuli were obtained by capturing the tristimulus values (CIE XYZ) of physical samples placed under diffuse illumination equivalent to D65 using a two-dimensional spectroradiometer (SR-5100, TechnoOptis Corp., Tokyo, Japan). RGB images were generated using a matrix that converts XYZ values to linear RGB, obtained through prior display calibration, and a look-up table (LUT) that converts linear RGB values to 16-bit RGB values while accounting for display gamma. The images were displayed on a custom calibrated monitor (ColorEdge CG3146, EIZO Corp., Ishikawa, Japan). The display was first calibrated using the built-in calibration function, and then a calibration LUT was created by custom calibration, which measured tristimulus values XYZ by displaying primary color RGB images and images with stepwise changes in pixel values.

To verify color reproduction accuracy, an X-Rite ColorChecker was imaged, and the XYZ values of the 24 color patches for both the physical chart and the displayed image were measured using a spectroradiometer (CS-2000, Konica Minolta, Inc., Tokyo, Japan). The mean color difference was CIE 1976 ΔE*ab ≤ 1.2. This value is approximately half of the color discrimination threshold of 2.3 reported by Mahy et al. [20], indicating that the CRIs achieved sufficient color reproduction accuracy.

**Figure 2:**
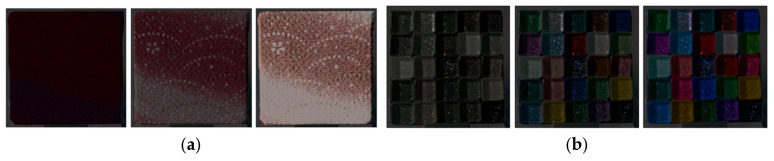
(**a**) Examples of lightness-modulated images for a fabric sample (from left to right: *k* = 0.1, 1.0, and 3.0). (**b**) Examples of chroma-modulated images for a glass sample (from left to right: *k* = 0.1, 1.0, and 3.0).

Subsequently, two types of modulated image sets were generated based on the CRI. The RGB values of the CRI were converted to tristimulus values using the calibrated conversion matrix and LUT, and CIE 1976 L*a*b* and CIE 1976 L*C*h values were computed. Multiplicative scaling with a factor *k* (ranging from 0.1 to 3.0 in increments of 0.1) was applied to L* or C*. The modified tristimulus values were then converted back to RGB values to generate the lightness-modulated images for Experiment A and the chroma-modulated images for Experiment B. Figure 2 respectively shows the examples of modulated images for Experiments A and B.

### 2.3. Measurement of Real Stimuli and Calculation of Image Features

Surface roughness parameters and GLCM features were used as quantitative indices for evaluating the physical and image stimuli. Surface roughness measurements were obtained by acquiring the surface height distribution of each physical sample using a 3D shape-measuring machine (VR-5100, Keyence Corp., Osaka, Japan). From the measured height distribution, six surface roughness parameters defined by ISO 25178 (Geometrical Product Specifications (GPS)—Surface texture: Areal) were calculated: arithmetic mean height ($S_{a}$), maximum height ($S_{z}$), ten-point height ($S_{10z}$), root mean square height ($S_{q}$), skewness ($S_{sk}$), and kurtosis ($S_{ku}$) [21]. These parameters correspond to the height specifications outlined in ISO 25178 and quantify the magnitude of surface irregularities, height variation, and the shape of the height distribution.

Next, the computation of image features is described. For the CRIs and the lightness- and chroma-modulated images, GLCMs were constructed based on the definition proposed by Haralick et al. [22]. A GLCM is a matrix that counts the number of occurrences in which a pixel with luminance value i is separated by a distance r and angle θ from a pixel with luminance value j. In this study, the number of gray levels was set to $2^{16}$, with r=1 and θ=0. The distance r=1 evaluates the luminance relationship between adjacent pixels and is a common method for capturing and analyzing minute luminance fluctuations and local texture structures on material surfaces. Furthermore, θ=0 is a fundamental direction commonly used in texture analysis and is adopted as a representative direction in this study. On the other hand, GLCM features are known to depend on parameter settings, and this study limits analysis to specific settings. However, since the objective of this study is to compare results under the same conditions, the impact of differences in parameter settings is limited. Therefore, this configuration maximizes the number of pixel pairs considered while capturing luminance variations between adjacent pixels at the shortest distance. To ensure that the resulting GLCM values were not dependent on the chosen distance, angle, or analysis region, the matrix was normalized by the total number of elements so that the sum of all entries equaled 1, forming a probability matrix.

From the normalized matrix, five image features were computed: contrast, dissimilarity, homogeneity, Angular Second Moment (ASM), and entropy. Contrast represents local luminance variation, dissimilarity represents image non-uniformity, homogeneity represents similarity, ASM represents regularity, and entropy represents complexity. Previous studies have suggested that these features may influence material perception, including glossiness and roughness [7,17]. As these features have different numerical scales, each was normalized so that its maximum possible value was 1. Specifically, each feature value was divided by its theoretical maximum derived from the number of gray levels. In this study, the number of gray levels was $2^{16}$, and the maximum values of each feature were defined based on this configuration. Contrast was normalized by ${\left(2^{16}\right)}^{2}$, dissimilarity by $2^{16}$, homogeneity and ASM were inherently bounded within [0, 1], and entropy was normalized by log ${\left(2^{16}\right)}^{2}$. Through this normalization, all features were scaled to a comparable range of [0, 1], enabling quantitative comparison across different image features.

### 2.4. Procedure

Observers directly and independently compared the physical object with each of the two types of modulated image sets and selected three images perceived to match the glossiness, transparency, and roughness of the physical object (designated as glossiness-reproduced image, transparency-reproduced image, and roughness-reproduced image, collectively referred to as appearance-reproduced images). To ensure equivalence of retinal image size between the physical object and the image, both stimuli were arranged to subtend a visual angle of 5.5°, making them appear identical in size. Under the present viewing conditions, the observed image resolution of the image stimuli was approximately 65 cycles per degree, allowing observers to view the images at a resolution equivalent to that of the physical objects.

**Figure 3:**
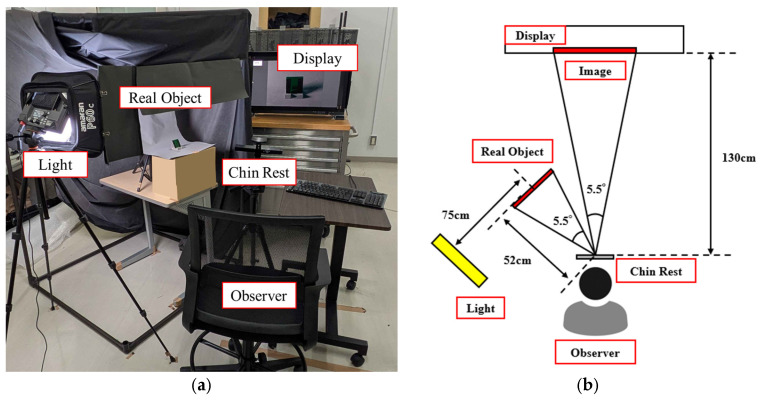
(**a**) Photograph of the experimental setup; (**b**) Schematic representation of the experimental configuration. Observers simultaneously viewed the real object and the colorimetrically reproduced image displayed on a calibrated monitor under controlled illumination conditions. The viewing geometry was adjusted so that both stimuli subtended the same visual angle of 5.5°. The distances between the observer, display, and real object are indicated in the schematic, ensuring equivalent retinal image size for comparison.

The procedure followed a method-of-adjustment matching paradigm [23,24,25] to achieve perceptual equivalence between physical objects and images (Figure 3). Following a 5 min adaptation, observers binocularly viewed stimuli side-by-side with matched retinal sizes (5.5° visual angle) and high spatial resolution (65 cpd) to ensure physical comparability and minimize biases. Using a keyboard, observers selected three appearance-reproduced images per sample for both lightness- and chroma-modulated sets. To ensure statistical reliability and intra-observer consistency, 15 observers evaluated all 55 samples twice. This sample size and the experimental design meet the requirements of Recommendation ITU-R BT.500-15 [26]. This procedure was performed separately for the lightness-modulated and chroma-modulated image sets.

## 3. Results

Fifteen observers in their twenties (22.0 ± 1.0 years), all with normal color vision and visual acuity of 1.0 or higher checked by the Ishihara Plates test, participated in the experiment. For each image feature of the selected appearance-reproduced images, the Smirnov–Grubbs test was applied to determine whether the value showing the largest deviation from the mean was an outlier (*p* < 0.05). When an outlier was detected, the procedure was repeated until no outliers remained. The mean value of each image feature was then calculated. To verify variability in ratings among observers, we calculated mean inter- and intra-observer variances based on luminance and chroma modulation magnitude (multiplicative scaling) in images selected by observers. For both experiments, the mean intra-observer variance was smaller than the mean inter-observer variance. This confirms that selections made by the same observer were generally reproducible. Although variability was observed among observers, each observer’s selections were relatively consistent. The mean values used in this study confirmed similar perceptual tendencies among observers.

### 3.1. Experiment A

To determine whether the image features of the appearance-reproduced images differed significantly from those of the CRI, paired *t*-tests were conducted (*p* < 0.05). Cohen’s effect size *d* was also calculated to evaluate the magnitude of the differences. Subsequently, image features were deemed to exhibit substantial change when both *p* < 0.05 and *d* ≥ 0.8 (large effect).

Stimuli showing significant differences between the CRI and the appearance-reproduced images were classified as Group 1, while those without significant differences were classified as Group 2. Differences in physical characteristics between groups were analyzed using the Mann–Whitney U-test. The physical characteristics examined included six surface roughness parameters ($S_{a}$, $S_{z}$, $S_{10z}$, $S_{q}$, $S_{sk}$, $S_{ku}$) derived from the surface height distribution of the physical objects, GLCM features of the CRI (contrast, dissimilarity, homogeneity, ASM, entropy), and color features (lightness, chroma).

#### 3.1.1. Glossiness

**Figure 4:**
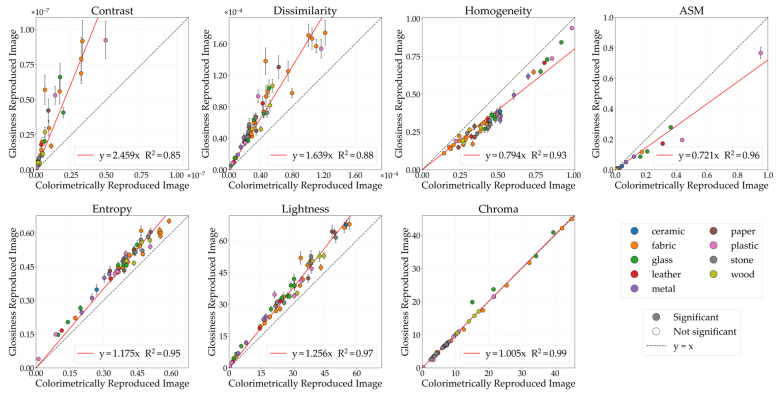
Comparison of image features between colorimetrically reproduced images (CRIs) and glossiness-reproduced images. Each point represents the mean value across observers. Error bars denote the standard error. Data points are color-coded according to material category. Filled circles (●) indicate stimuli showing significant differences, whereas open circles (○) indicate non-significant stimuli, based on paired *t*-tests (*p* < 0.05) and Cohen’s effect size (*d* ≥ 0.8).

Figure 4 illustrates the comparative analysis of GLCM features (contrast, dissimilarity, homogeneity, ASM, entropy) and color features (lightness, chroma) between the CRI and the glossiness-reproduced images selected by observers. The horizontal axis represents the CRI values, while the vertical axis indicates the glossiness values of the reproduced images. Each data point corresponds to the mean image feature value, while the error bars denote the standard error. The black diagonal line represents *y* = *x*; stimuli located on this line indicate no change in image features between the CRI and the glossiness-reproduced images. The colored lines represent regression lines constrained to pass through the origin. Data points are color-coded according to material category. Filled circles (●) indicate stimuli showing significant differences, whereas open circles (○) indicate non-significant stimuli between the CRI and the glossiness-reproduced images. Although the approximation accuracy improved with polynomial fit, problems such as decreased interpretability also arise, so in this study, we discussed based on a linear fit with the aim of concisely showing the relationship between physical quantities and image features.

**Figure 5:**
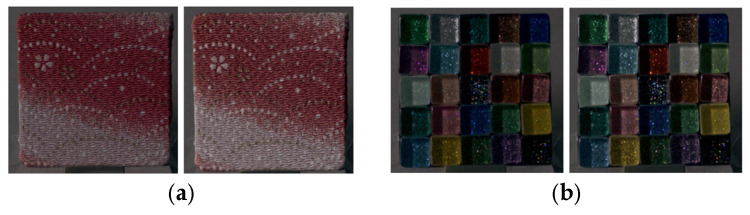
(**a**) CRI and the image with lightness increased by a factor of 1.3 (fabric sample); (**b**) CRI and the image with lightness increased by a factor of 1.3 (glass sample).

The lightness of the glossiness-reproduced images was 1.26 times that of the CRIs. Because lightness and chroma modulation were performed in increments of 0.1, no image corresponds exactly to a factor of 1.26. Therefore, Figure 5 presents the lightness-modulated image with the closest factor, 1.3. With lightness modulation, contrast, dissimilarity, and entropy increased, whereas homogeneity and ASM decreased in the glossiness-reproduced images. Moreover, contrast, dissimilarity, homogeneity, entropy, and lightness changed in the same direction across all 55 stimuli.

These results suggest that increasing image lightness may align perceived glossiness between physical objects and images regardless of material category or physical characteristics. Previous studies [4,16,17] have reported that glossiness perceived from images can be weaker than that of physical objects. Possible causes include differences in binocular disparity between three-dimensional objects and two-dimensional images and reduced perceptibility of local luminance differences in images. These factors are considered to occur irrespective of the viewing environment. Therefore, in this study, glossiness perceived from images was weaker than that of physical objects, and increasing image lightness may have enhanced inter-pixel luminance differences and luminance-distribution complexity, compensating for the reduction in perceived glossiness associated with image reproduction.

**Figure 6:**
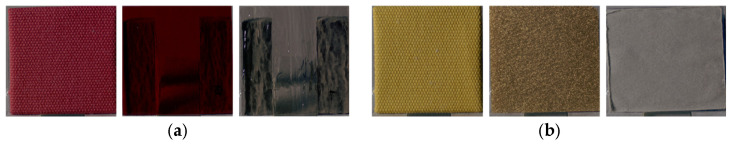
(**a**) Group 1 samples (fabric, glass, glass). (**b**) Group 2 samples (fabric, plastic, paper).

Next, differences in physical characteristics between Group 1 (stimuli showing significant changes in image features) and Group 2 (stimuli showing no significant changes) were examined using the Mann–Whitney U-test. According to ASM classification, 52 stimuli were assigned to Group 1 and 3 stimuli to Group 2. Representative examples of each group are shown in Figure 6. For dissimilarity, the mean ± standard deviation was 3.51 ± 2.69 for Group 1 and 7.70 ± 4.13 for Group 2 (p=0.039). For homogeneity, the values were 0.46 ± 0.19 for Group 1 and 0.25 ± 0.09 for Group 2 (p=0.024). Stimuli categorized as Group 2 had significantly higher dissimilarity and considerably lower homogeneity compared to those in Group 1. These stimuli already had large inter-pixel luminance differences and low similarity at the CRI stage; therefore, perceived glossiness between the physical object and the image could match without substantial reduction in ASM.

The present results suggest a relationship between glossiness perception and lightness perception, supporting previous findings that report associations between glossiness and perceived brightness [4,7,8]. This may be because image features related to light reflection, such as highlights and luminance contrast, serve as cues for gloss perception and are amplified by lightness modulation.

#### 3.1.2. Transparency

**Figure 7:**
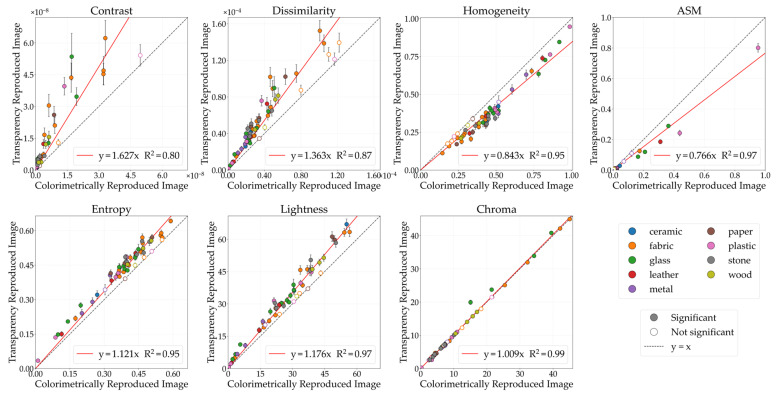
Comparative analysis of image features between CRI and transparency-reproduced image.

**Figure 8:**
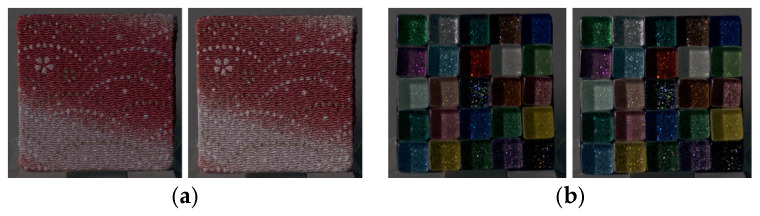
(**a**) CRI and the image with lightness increased by a factor of 1.2 (fabric sample); (**b**) CRI and the image with lightness increased by a factor of 1.2 (glass sample).

Figure 7 illustrates the comparative analysis of image features between the CRI and the transparency-reproduced images selected by observers. The lightness of the transparency-reproduced images was 1.18 times greater than that of the CRI. Because modulation was performed in increments of 0.1, Figure 8 presents the lightness-modulated image with a factor of 1.2, which is the closest condition. With lightness modulation, contrast, dissimilarity, and entropy increased, whereas homogeneity and ASM decreased in the transparency-reproduced images. Moreover, contrast, dissimilarity, homogeneity, ASM, entropy, and lightness changed in the same direction across all 55 stimuli.

**Figure 9:**
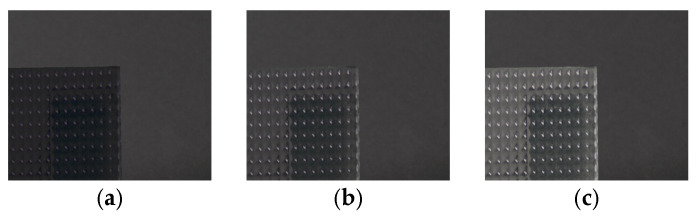
Differences in background visibility through a transparent object (glass sample with uniformly distributed surface irregularities). (**a**) Lightness-modulated image with lightness scaled to 0.5 relative to CRI shown in (**b**); (**b**) CRI; (**c**) lightness-modulated image with lightness scaled to 1.5 relative to CRI shown in (**b**).

Previous studies [16,17] have reported that transparency perceived from images can be weaker than that of physical objects. Similar to with glossiness, possible causes include differences in binocular disparity between three-dimensional objects and two-dimensional images and reduced perceptibility of local luminance differences in images. In addition, for transparency, the appearance of the background due to light transmission and scattering within the object may serve as an important cue. In the present image modulation method, as shown in Figure 9, the lightness of the stimulus region was altered, whereas the background region outside the stimulus remained constant across all images. For stimuli with translucent properties, increasing lightness enhanced the luminance contrast between the stimulus and the background, thereby emphasizing perceptual cues related to transmission and scattering. This improvement likely contributed to the alignment of perceived transparency between physical objects and images.

**Figure 10:**
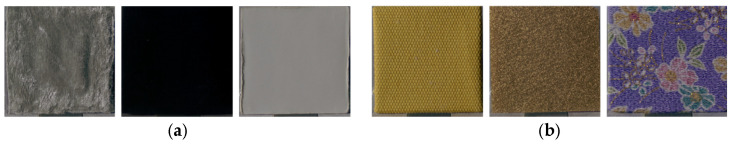
(**a**) Group 1 samples (fabric, plastic, paper). (**b**) Group 2 samples (fabric, plastic, fabric).

Next, differences in physical characteristics between Group 1 (stimuli showing significant changes in image features) and Group 2 (stimuli showing no significant changes) were examined using the Mann–Whitney U-test. Table 1 presents the mean and standard deviation of the physical characteristics for groups classified based on dissimilarity, homogeneity, and lightness. Representative stimuli for each group are shown in Figure 10. Stimuli classified into Group 1 exhibited significantly lower CRI contrast, dissimilarity, and chroma, and significantly higher homogeneity, compared with those in Group 2. Transparency perceived from images tends to be weaker than that of physical objects, and this tendency was particularly pronounced for stimuli characterized by small inter-pixel luminance differences, low chroma, and high similarity in the CRI. One possible explanation is that when observing physical objects, binocular disparity provides depth cues and variations in surface appearance, whereas in images, pixel relationships are fixed. When chroma and local luminance variations are small, depth cues derived from pixel differences may be reduced. For such stimuli, enhancing lightness increased local luminance differences and luminance contrast, allowing images to attain perceptual transparency comparable to that of physical objects.

These findings also suggest that transparency perception between physical objects and images may rely not only on background visibility through transparent materials but also on integrated appearance information, encompassing surface luminance variations and chromatic properties of both transparent and opaque objects.

#### 3.1.3. Roughness

**Figure 11:**
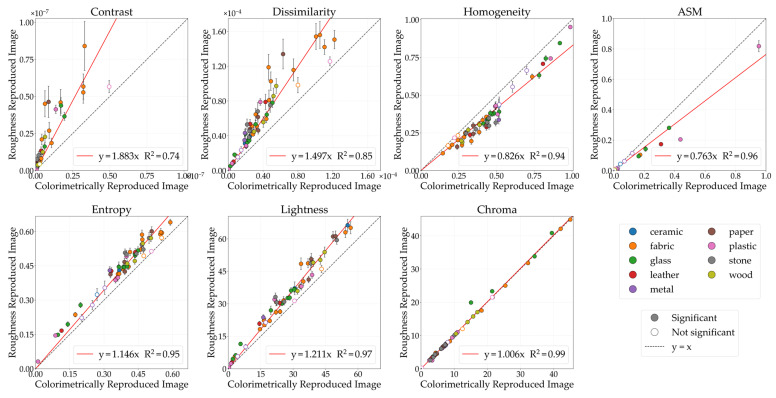
Comparative analysis of image features between CRI and the roughness-reproduced image.

Figure 11 illustrates the comparative analysis of image features between the CRIs and the roughness-reproduced images selected by observers. The lightness of the roughness-reproduced images was 1.21 times greater than that of the CRIs. Because modulation was performed in increments of 0.1, Figure 8 presents the lightness-modulated image with a factor of 1.2, which is the closest condition. In the roughness-reproduced images, lightness modulation resulted in increased contrast, dissimilarity, and entropy, whereas homogeneity and ASM decreased. Moreover, contrast, dissimilarity, homogeneity, ASM, entropy, and lightness changed in the same direction across all 55 stimuli.

Previous studies [16,17] have reported that roughness perceived from images can be either stronger or weaker than that of physical objects. For stimuli with complex surfaces or large surface irregularities, roughness in images may be perceived as weaker when image reproduction fails to fully capture those characteristics. In contrast, for stimuli lacking complex surface structures, roughness may be perceived as stronger due to sampling effects introduced during image digitization. In addition, observers selected images perceived as having roughness equivalent to that of physical objects from a set of CRIs whose spatial frequency characteristics were systematically modulated. That result demonstrated that a decrease in ASM was effective for reproducing perceived roughness, suggesting that roughness of physical objects can be reproduced in images by adjusting image features to reduce image regularity.

The current results indicate that increasing lightness enhanced contrast and dissimilarity, which represent local luminance differences, as well as entropy, which reflects image complexity, while reducing homogeneity and ASM, which represent similarity and regularity. These findings support the results of previous studies. These changes in image features likely emphasized shading patterns caused by surface irregularities, thereby enabling the reproduction of perceived roughness in images. The present results further indicate that roughness perception does not depend solely on the faithful reproduction of physical surface geometry, but is strongly influenced by perceptual cues based on luminance distribution. In particular, enhancement of local luminance differences through lightness modulation appears to be an effective operation for reproducing perceived roughness in images.

**Figure 12:**
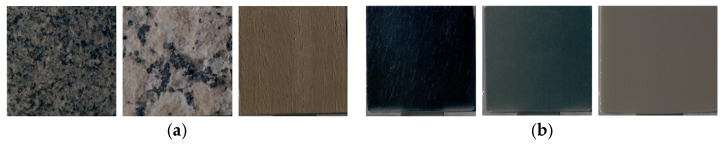
(**a**) Group 1 samples (stone, stone, wood). (**b**) Group 2 samples (metal, metal, ceramic).

Next, differences in physical characteristics between Group 1 (stimuli showing significant changes in image features) and Group 2 (stimuli showing no significant changes) were examined using the Mann–Whitney U-test. Table 2 presents the mean and standard deviation of the physical characteristics for groups classified based on homogeneity, ASM, entropy, and lightness. Representative stimuli for each group are shown in Figure 12. Stimuli categorized as Group 1 exhibited significantly higher surface roughness parameters of the physical objects ($S_{a}$, $S_{z}$, $S_{10z}$, $S_{q}$) in contrast to those in Group 2. For stimuli with large surface roughness, observation of the physical object provides height and depth information derived from surface irregularities, along with fine shading cues resulting from micro-scale relief, both of which contribute to roughness perception. In contrast, when observing images, some of these cues are reduced, and roughness must be judged based on planar representations of surface structure and shading.

By increasing image lightness, the brightness of darker regions was enhanced, highlighting fine surface irregularities and shading cues that were otherwise difficult to perceive in the image. Furthermore, decreases in homogeneity and ASM, along with increases in entropy, signify heightened complexity in the luminance structure of the image. These changes presumably contributed to aligning perceived roughness between the physical objects and the images.

### 3.2. Experiment B

The same fifteen observers who participated in Experiment A also took part in Experiment B. The data were analyzed using the same procedures as in Experiment A.

#### 3.2.1. Glossiness

**Figure 13:**
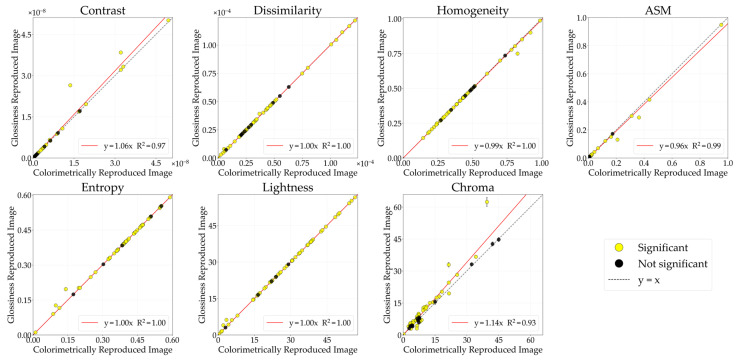
Comparative analysis of image features between CRIs and the glossiness-reproduced image.

**Figure 14:**
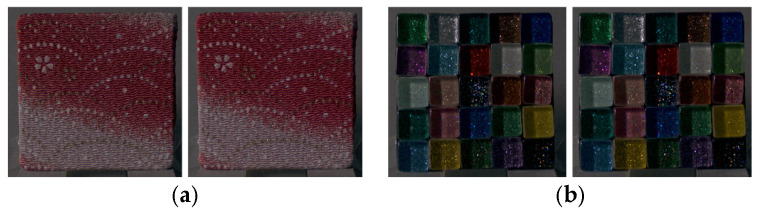
(**a**) CRI and the image with chroma increased by a factor of 1.1 (fabric sample); (**b**) CRI and the image with chroma increased by a factor of 1.1 (glass sample).

Figure 13 illustrates the comparative analysis of image features between the CRIs and the glossiness-reproduced images selected by observers. The chroma of the glossiness-reproduced images was 1.14 times that of the CRIs. Because modulation was performed in increments of 0.1, Figure 14 presents the chroma-modulated image with a factor of 1.1, which is the closest condition. Overall, the changes in GLCM features induced by chroma modulation were small. This is likely because chroma modulation does not alter luminance values, and therefore changes in adjacent-pixel luminance, as captured by the GLCM, were minimal.

**Figure 15:**
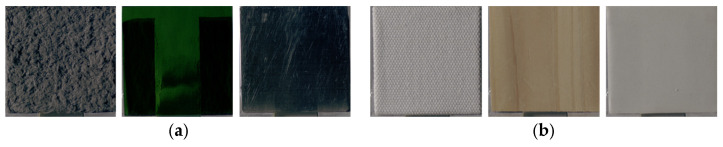
(**a**) Group 1 samples (fabric, glass, metal); (**b**) Group 2 samples (fabric, wood, paper).

Next, differences in physical characteristics between Group 1 (stimuli showing significant changes in image features) and Group 2 (stimuli showing no significant changes) were examined using the Mann–Whitney U-test. Representative stimuli for each group are shown in Figure 15. When grouping was performed based on ASM, 45 stimuli were categorized as Group 1 and 10 stimuli as Group 2. The lightness of the CRI was 24.70 ± 14.02 for Group 1 and 37.34 ± 15.06 for Group 2 (p=0.012), indicating that stimuli in Group 2 had significantly higher CRI lightness than those in Group 1. For stimuli with high lightness and bright surfaces, chroma modulation may have had limited influence on local luminance patterns within the luminance image, making reductions in ASM less detectable. In contrast, for stimuli with darker surface characteristics, decreasing ASM, which reflects image regularity, may have contributed to aligning perceived glossiness between the physical objects and the images.

#### 3.2.2. Transparency

**Figure 16:**
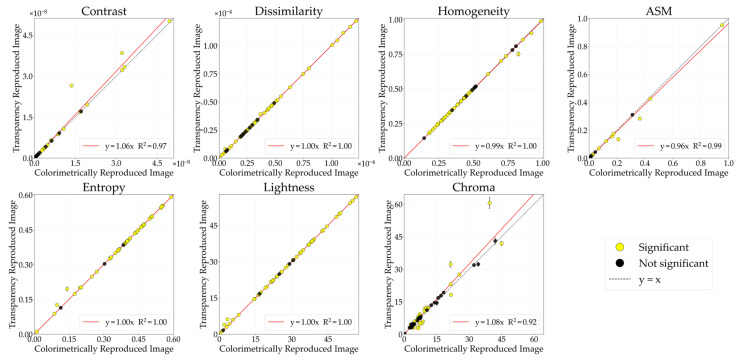
Comparative analysis of image features between CRI and the transparency-reproduced image.

Figure 16 illustrates the comparative analysis of image features between the CRIs and the transparency-reproduced images selected by observers. The chroma of the transparency-reproduced images was 1.08 times greater than that of the CRI. Overall, the changes in GLCM features resulting from chroma modulation were small.

**Figure 17:**
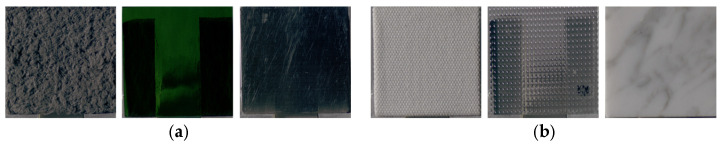
(**a**) Group 1 samples (fabric, glass, metal). (**b**) Group 2 samples (fabric, glass, stone).

Next, differences in physical characteristics between Group 1 (stimuli showing significant changes in image features) and Group 2 (stimuli showing no significant changes) were examined using the Mann–Whitney U-test. Representative stimuli for each group are shown in Figure 17. According to ASM classification, 46 stimuli were categorized as Group 1 and 9 stimuli as Group 2. The CRI lightness was 25.09 ± 14.14 for Group 1 and 36.77 ± 15.67 for Group 2 (p=0.024), indicating that stimuli in Group 2 had significantly higher CRI lightness compared to those in Group 1. This result is similar to that observed for glossiness. For stimuli characterized by lower surface lightness, reducing ASM, which reflects image regularity, may have contributed to aligning perceived transparency between the physical objects and the images.

#### 3.2.3. Roughness

**Figure 18:**
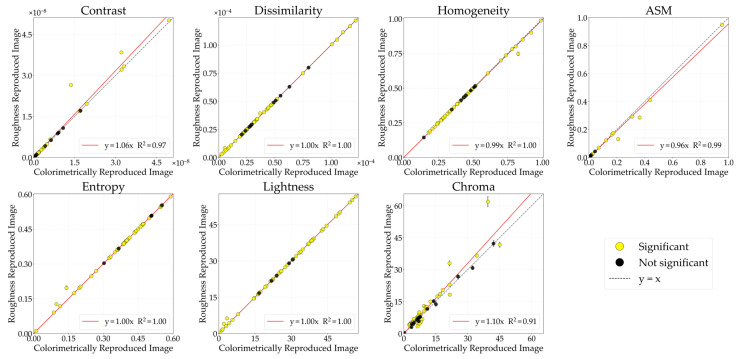
Comparative analysis of image features between CRI and the roughness-reproduced image.

Figure 18 illustrates the comparative analysis of image features between the CRIs and the roughness-reproduced images selected by observers. The chroma of the roughness-reproduced images was 1.10 times that of the CRIs. Overall, the changes in GLCM features induced by chroma modulation were small.

**Figure 19:**
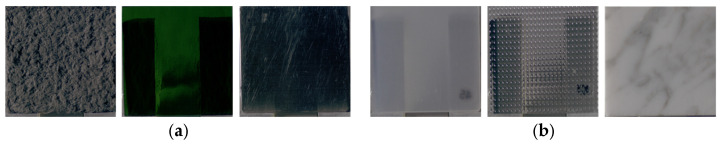
(**a**) Group 1 samples (fabric, glass, metal). (**b**) Group 2 samples (plastic, glass, stone).

Next, differences in physical characteristics between Group 1 (stimuli showing significant changes in image features) and Group 2 (stimuli showing no significant changes) were examined using the Mann–Whitney U-test. Representative stimuli for each group are shown in Figure 19. When grouping was performed based on ASM, 46 stimuli were classified into Group 1 and 9 stimuli into Group 2. The CRI lightness was 25.04 ± 15.29 for Group 1 and 37.03 ± 6.97 for Group 2 (p=0.013), indicating that stimuli in Group 2 had significantly higher CRI lightness than those in Group 1.

In Experiment B, across all three appearance attributes, grouping based on ASM consistently revealed that stimuli in Group 2 were characterized by lower CRI lightness. These results suggest that the regularity of the luminance distribution may serve as a cue for material perception. For stimuli with low lightness, increasing the complexity of the luminance distribution by reducing its regularity may facilitate the reproduction of perceived material appearance in images.

While these results demonstrate the effectiveness of lightness and chroma modulation, it is important to consider the inherent limitations of using static 2D images. The observed discrepancies in material appearance might also stem from the lack of three-dimensional (3D) information. Previous studies have shown that binocular disparity and motion parallax significantly enhance glossiness perception [27]. Furthermore, binocularly decorrelated highlights, known as “proto rivalry,” are critical cues for the visual system to identify specular reflections [28], while surface opacity perception is strongly coupled with the interpretation of 3D geometry [29]. These factors suggest that 2D representations have fundamental limits in replicating the full material appearance of real-world objects. Recent advancements in computer graphics and HDR imaging have focused on optimizing material appearance by incorporating these perceptual factors [14] and developing advanced assessment metrics for rendering quality [30]. Future research should explore how the modulations identified in this study interact with such dynamic and 3D cues.

## 4. Conclusions

In this study, the problem that perceived material appearance is not necessarily equivalent between physical objects and CRIs was addressed. The objective was to clarify the conditions under which equivalent material appearance is perceived between physical objects and images. Image sets were generated by independently modulating the lightness and chroma of the CRI, and the effects of these modulations on perceived glossiness, transparency, and roughness were analyzed. In the experiments, observers directly compared physical objects with lightness- and chroma-modulated images, selecting images they perceived as corresponding to each appearance attribute.

In Experiment A, lightness-modulated images chosen for all attributes required higher lightness than the CRIs. This modulation increased contrast, dissimilarity, entropy, and lightness, while decreasing homogeneity and ASM. These results suggest that observers rely on local luminance variations to compensate for the lack of 3D cues—such as binocular disparity and surface geometry—inherent in physical objects. Enhancing image lightness likely restores luminance complexity, enabling perceptual matching by emulating object-specific cues.

In Experiment B, selected images exhibited increased chroma with minimal changes in GLCM features. This indicates that chroma itself serves as a perceptual cue, potentially through the Helmholtz–Kohlrausch effect, where higher chroma enhances subjective brightness. Thus, chroma modulation may facilitate material perception independently of image statistics.

Across both experiments, Group 1 stimuli (requiring significant feature modifications) initially lacked contrast and lightness compared to Group 2 at the CRI stage. This confirms that larger adjustments are necessary when the CRI fails to preserve essential luminance differences. Specifically, for roughness in Experiment A, Group 1 corresponded to objects with higher physical surface roughness. For such objects, increasing luminance complexity and emphasizing shading cues (via changes in homogeneity and ASM) effectively compensates for the absence of physical depth information.

The main contribution of this research lies in quantitatively demonstrating that modulation of lightness and chroma is effective in reproducing glossiness, transparency and roughness, which has been difficult with conventional color reproduction (CRI). This finding is expected to have applications in future image processing algorithms, such as accurate appearance display in e-commerce and appearance and quality editing techniques in HDR displays. However, this study has several limitations. First, the experiments were conducted under controlled viewing conditions using a specific display environment, and therefore the generalizability of the findings to other viewing conditions or display systems remains uncertain. For example, this study uses a controlled darkroom environment and a high-definition LCD; however, different display technologies such as OLEDs, or different lighting conditions (such as bright light), may alter perceived contrast and saturation, potentially affecting the results. Second, only lightness and chroma modulations were examined based on a single CRI framework, and other image manipulations or rendering techniques may influence material perception differently. In addition, although the results suggest that luminance-based image statistics contribute to perceptual matching, causal relationships between specific image features and material perception were not directly manipulated in isolation. Further studies are needed to examine these factors across a broader range of materials, image generation methods, and observer populations.

Future work should focus on constructing predictive models that estimate the image features of appearance-reproduced images based on the physical characteristics of objects and the image features of the CRI. Such models must account for inter-observer variability and generalize across diverse materials and surface properties, requiring further experimental investigation. In addition, to analyze the causal relationship between changes in image features and perceptual coincidence, it is necessary to control and manipulate individual image features and isolate their effects.

## Figures and Tables

**Table 1:** Mean and standard deviation of physical characteristics for groups categorized by dissimilarity, homogeneity, and lightness. The values of contrast and dissimilarity were multiplied by 10^−9^ and 10^−5^, respectively.

Image Feature	Group	Contrast (×10^−9^)	Dissimilarity (×10^−5^)	Homogeneity	Chroma
Dissimilarity	Group 1 (49)	3.88±6.30	3.17±2.19	0.47±0.19	10.30±9.77
	Group 2 (6)	21.60±19.30	8.39±3.90	0.25±0.06	19.74±12.07
	*p* value	0.003	0.001	0.001	0.013
Homogeneity	Group 1 (48)	3.95±6.35	3.20±2.21	0.47±0.19	10.35±9.87
	Group 2 (7)	18.60±19.30	7.46±4.33	0.29±0.12	18.07±11.87
	*p* value	0.025	0.011	0.011	0.015
Lightness	Group 1 (48)	3.95±6.35	3.20±2.21	0.47±0.19	10.35±9.87
	Group 2 (7)	18.60±19.30	7.46±4.33	0.29±0.12	18.07±11.87
	*p* value	0.025	0.011	0.011	0.015

**Table 2:** Mean and standard deviation of physical characteristics for groups categorized by homogeneity, ASM, entropy, and lightness.

Image Feature	Group	*S_a_* [*μm*]	*S_z_* [*μm*]	*S*_10*z*_ [*μm*]	*S_q_* [*μm*]
Homogeneity	Group 1 (48)	0.12±0.11	1.50±1.66	0.73±0.92	0.15±0.15
	Group 2 (7)	0.06±0.06	0.45±0.41	0.20±0.18	0.07±0.07
	*p* value	0.040	0.045	0.015	0.033
ASM	Group 1 (42)	0.12±0.11	1.61±1.73	0.79±0.96	0.16±0.15
	Group 2 (13)	0.06±0.06	0.55±0.55	0.26±0.27	0.08±0.08
	*p* value	0.010	0.006	0.003	0.007
Entropy	Group 1 (48)	0.12±0.11	1.50±1.66	0.73±0.92	0.15±0.15
	Group 2 (7)	0.06±0.06	0.45±0.41	0.20±0.18	0.07±0.07
	*p* value	0.040	0.045	0.015	0.033
Lightness	Group 1 (49)	0.11±0.11	1.48±1.64	0.73±0.91	0.15±0.15
	Group 2 (6)	0.05±0.06	0.41±0.44	0.17±0.17	0.06±0.07
	*p* value	0.024	0.039	0.008	0.021

## Data Availability

The data presented in this study are available on request from the corresponding author due to restrictions.

## Abbreviations

The following abbreviations are used in this manuscript:

CRI	colorimetrically reproduced image
GLCM	gray-level co-occurrence matrix
DOI	distinctness-of-image
GU	gloss units
LUT	look-up table
ASM	Angular Second Moment
